# Altered Mitochondrial Dynamics in Motor Neuron Disease: An Emerging Perspective

**DOI:** 10.3390/cells9041065

**Published:** 2020-04-24

**Authors:** Manohar Kodavati, Haibo Wang, Muralidhar L. Hegde

**Affiliations:** 1 Department of Neurosurgery, Center for Neuroregeneration, Houston Methodist Research Institute, Houston, TX 77030, USA; mkodavati@houstonmethodist.org (M.K.); hwang@houstonmethodist.org (H.W.); 2 Department of Neurosurgery, Weill Medical College, New York, NY 10065, USA

**Keywords:** mitochondria, FUS, motor neuron disease, amyotrophic lateral sclerosis, frontotemporal dementia, DNA damage, neurodegeneration

## Abstract

Mitochondria plays privotal role in diverse pathways that regulate cellular function and survival, and have emerged as a prime focus in aging and age-associated motor neuron diseases (MNDs), such as amyotrophic lateral sclerosis (ALS) and frontotemporal dementia (FTD). Accumulating evidence suggests that many amyloidogenic proteins, including MND-associated RNA/DNA-binding proteins fused in sarcoma (FUS) and TAR DNA binding protein (TDP)-43, are strongly linked to mitochondrial dysfunction. Animal model and patient studies have highlighted changes in mitochondrial structure, plasticity, replication/copy number, mitochondrial DNA instability, and altered membrane potential in several subsets of MNDs, and these observations are consistent with the evidence of increased excitotoxicity, induction of reactive oxygen species, and activation of intrinsic apoptotic pathways. Studies in MND rodent models also indicate that mitochondrial abnormalities begin prior to the clinical and pathological onset of the disease, suggesting a causal role of mitochondrial dysfunction. Our recent studies, which demonstrated the involvement of specific defects in DNA break-ligation mediated by DNA ligase 3 (LIG3) in FUS-associated ALS, raised a key question of its potential implication in mitochondrial DNA transactions because LIG3 is essential for both mitochondrial DNA replication and repair. This question, as well as how wild-type and mutant MND-associated factors affect mitochondria, remain to be elucidated. These new investigation avenues into the mechanistic role of mitochondrial dysfunction in MNDs are critical to identify therapeutic targets to alleviate mitochondrial toxicity and its consequences. In this article, we critically review recent advances in our understanding of mitochondrial dysfunction in diverse subgroups of MNDs and discuss challenges and future directions.

## 1. Introduction

Aging-associated neurological disorders are considered the foremost public health challenge of our time, having devastating effects on quality of life and forming a major burden on health-care systems. Motor neuron diseases (MNDs) are one such group of progressive neurodegenerative disorders characterized by dysfunction of lower motor neurons in the ventral horn and/or upper motor neurons in the precentral gyrus of the spinal cord. This typically results in loss of control over voluntary muscle activities such as walking, speaking, breathing, and swallowing. MNDs are predominantly sporadic with multifactorial etiology, but a smaller subset of ~5–10% can be familial, involving one or more mutations or genetic factors usually of autosomal dominant inheritance [1]. The incidence of motor neuron disease in the U.S. is approximately 3–5 cases for 100,000 population per year, and the average time from diagnosis to death is about 3 years [2].

Amyotrophic lateral sclerosis (ALS) and frontotemporal dementia (FTD; also known as frontotemporal lobar dementia or FTLD) are the two major MNDs, with both overlapping and unique etiologies and clinical features. Notably, the disease name defines the important features of the disease. Amyotrophy refers to atrophy of muscle fibers, which leads to weakness in affected muscles and observable fasciculations. Lateral sclerosis refers to the hardening of lateral and anterior corticospinal tracts, as motor neurons within these areas degenerate and are replaced by gliosis. Unlike ALS, FTD involves a progressive neuronal atrophy with loss of frontal and temporal cortices, which is characterized by changes in personality and behavior, and gradual impairment of language skills. In ALS, familial and sporadic cases account for ~10% and ~90%, respectively, whereas in FTD, a strong genetic contribution is reflected by the higher percentage (50%) of patients having familial history [3]. The overlapping existence of ALS and FTD has been observed in many patients; about 15% of patients with FTD develop ALS and 5–22% of patients with ALS develop FTD (refered here as ALS-FTD) [4,5,6]. Patients with ALS-FTD generally have early onset symptoms in their 50s, similar to ALS without dementia, and it is more common in men than in women [7]. ALS symptoms may precede, occur simultaneously with, or follow the signs of FTD, although more frequently, cognitive changes precede motor weakness [8,9]. The interval between cognitive symptoms and weakness can range from months up to 7 years, with an average of 2 years [10]. ALS-FTD is normally more aggressive in comparison to either disease alone. Survival post-diagnosis varies from 2 to 5 years in ALS, whereas it is ≤2 years for ALS-FTD. Notably, ALS patients with dysexecutive function have worse survival, whereas those with abnormalities that are limited to language or visuospatial skills have comparable survival to cognitively normal ALS patients. ALS patients with dementia are mainly characterized by poor memory and cognitive defects, similar to Alzheimer’s disease (AD); however, this does not seem to affect overall survival [3].

Multiple environmental and genetic factors have been identified to contribute to ALS incidence and disease progression. Some putative environmental risk factors for ALS are history of brain trauma, radiation exposure, electrical shocks, smoking in the case of post-menopausal women, dietary factors, diabetes, physical fitness, viruses, and metal exposure [11,12,13,14,15,16,17]. The role of exogenous risk factors in ALS is reported inconsistently, indicating that these risk factors do not always cause pathology; instead, they reflect the potential interaction between various environmental factors and specific gene susceptibilities.

ALS was initially thought to be a sporadic disease [18]; however, evidence of the autosomal dominant inheritance of ALS was observed in subsequent studies, leading to associations with more than 100 genes [19]. Various bioinformatics repositories are available that list known ALS and FTD genes [20,21]. Many of these genes are, however, considered to be ‘minor genes’ because they have poor penetrance. Major genes that have been reported in multiple subsets of ALS or FTD include superoxide dismutase 1 (*SOD1*), TAR DNA binding protein (*TARDBP* coding for TDP-43 protein), fused in sarcoma (*FUS*), chromosome 9 open reading frame 72 (*C9ORF72*), microtubule-associated protein tau (*MAPT*), progranulin (*PGRN*), and valosin containing protein (*VCP*). C9ORF72 hexanucleotide repeat expansion is the most common cause of familial FTD and ALS, associated with ~30–50% of familial ALS, 5–7% of sporadic, ALS, 25% of familial FTD, and 6% of sporadic FTD [22,23]. Repeat expansion of the hexanucleotide repeat GGGGCC (G4C2) in the range of several hundred to thousands of repeats is seen in patients, in comparison to between two and 10 for the majority of the population [24,25]. The *SOD1* gene, which encodes the Cu-Zn superoxide dismutase, is one of three proteins involved in the conversion of free superoxide radicals to molecular oxygen and hydrogen peroxide. Mutations in the *SOD1* gene are found in 10–20% of familial ALS cases and 1–5% of sporadic ALS cases globally [26]; so far, more than 170 mutations of the *SOD1* gene are known in ALS. However, SOD1 knockout in rodents does not cause the disease phenotype, suggesting that the pathogenicity does not involve loss of function, and rather involves gain of toxic function, which can be due to the formation of aggregates caused by protein instability [27,28]. Mutations in FUS have been identified in nearly 5% of familial ALS patients along with 1% of sporadic ALS cases and about 10–20% of familial FTD [29,30]. Most mutations in FUS are clustered in the C-terminal nuclear localization sequence (NLS), thus inducing nuclear depletion and cytosolic aggregation. FUS binds both RNA and DNA and plays roles in RNA metabolism and maintaining genome integrity by being involved in the DNA damage response [31,32,33]. Pathogenic inclusions of FUS without TDP-43 or Tau containing aggregates are present in about 10% of FTLD cases, also known as FTLD-FUS [34]. Similar to FUS, TDP-43 is an RNA/DNA-binding protein known to function in RNA metabolism and transport of subcellular RNA. In healthy neurons, TDP-43 is localized to the nucleus, whereas toxic or mutated TDP-43 is cleaved and phosphorylated abnormally, and accumulated in ubiquitinated cytoplasmic inclusions in ALS [35]. More than 30 mutations are reported in the *TARDBP* gene with ~4–5% familial and ~2% sporadic ALS association [36]. MAPT mutations are associated with ~10–30% of familial FTD and normally occur together with TDP-43 and other pathology [37]. The *MAPT* gene encodes a 758-amino acid-long Tau protein, which is important for the binding and stabilization of microtubules located in neuronal axons. The mutated Tau protein becomes hyperphosphorylated and accumulates as abnormal filaments within neuronal and glial cells [38]. The *PGRN* gene encodes a precursor of granulin. PGRN is a growth factor involved in various metabolic events such as wound healing, tumor growth, and inflammation. PGRN also activates several kinase-dependent signaling cascades involved in controlling the cell cycle and motility [39]. In the United States, ~10% of FTD cases carry a mutation in *PGRN* gene, among which ~22% are familial [40]. Immunohistochemical studies show an increase in PGRN expression with disease progression in the spinal cords of transgenic animals with MNDs, and reveal strong expression of PGRN in the microglia of ALS patients [41,42,43].

Despite the association of multiple genes with ALS and FTD in the past decade, the precise etiology and mechanism of disease progression remain elusive. Defects in oxidative phosphorylation, calcium (Ca^2+^) buffering, and mitochondrial transport, normally seen at disease onset in the majority of patients, suggests the involvement of mitochondrial dysfunction in the etiology of ALS. In addition to their role as energy producers, mitochondria play a central role in Ca^2+^ homeostasis, phospholipid biogenesis, and apoptosis. Mitochondrial function is particularly crucial in the brain; despite constituting only 2% of body mass, the brain consumes 20% of the body’s resting ATP production. In neurotransmission, mitochondria act as essential Ca^2+^ buffering organelles. Due to their long life span, neurons may be more susceptible to damage caused by mitochondrial dysfunction. Furthermore, many proteins linked to ALS and FTD, including SOD1, TDP-43, FUS, and C9ORF72, are shown to interact with mitochondria. The association of these proteins with mitochondria is emerging as a critical factor in triggering disease onset and progression.

This review focuses on the central role of mitochondria in MNDs, the implications of recent findings regarding the role of TDP-43 and FUS in DNA repair and how they may play a critical role in the maintenance of genome stability in mitochondria.

## 2. Role of Mitochondrial Function and Plasticity in Central Nervous System (CNS)

The mitochondria are sites of aerobic respiration and supply energy for cellular needs in the form of ATP. Apart from ATP production, mitochondria are involved in a myriad of other cellular processes including amino acid and nucleotide metabolism, protein synthesis, fatty acid metabolism, ion homeostasis, and apoptosis [44,45]. Maintenance of mitochondrial quality control is important for cell survival. A mitochondrion has its own proteolytic system, which degrades misfolded/toxic proteins [46]. Damaged proteins on the outer mitochondrial membrane (OMM) are degraded by proteosome [47]. These multiple quality control mechanisms cumulatively help in maintaining healthy mitochondrial homeostasis. In addition, the fission and fusion processes within and among mitochondria help in clearing/redistribution of damaged components [48]. The mitochondrial respiratory chain, comprised of four multienzyme complexes designated as NADH coenzyme Q reductase (Complex I), succinate coenzyme Q reductase (Complex II), ubiquinol cytochrome c reductase (Complex III), and cytochrome c oxidase (Complex IV), is localized in the inner mitochondrial membrane (IMM) along with two electron carriers, cytochrome C and ubiquinone [49]. The mammalian mitochondrial proteome is comprised of ~1200 proteins, the majority of which are expressed from the nuclear genome, whereas a small subset of these proteins are expressed by the heritably and spatially separate mitochondrial genome [50]. Human mitochondrial DNA encodes 11 mRNAs, 2 rRNAs, and 22 tRNAs [51]. In higher metazoans, the mitochondrial genome is attached firmly to the IMM, packaged into DNA-protein complexes with mitochondrial transcription factor A (TFAM) [52].

Energy production and Ca^2+^ homeostasis maintained by mitochondria are particularly important in the CNS due to the high metabolic activity and energy needs of neurons. Local Ca^2+^ concentrations are critical for inter-neuronal communications [53]. Notably, bioenergetics, Ca^2+^ transport, and redox homeostasis in mitochondria form a tight network and these processes influence each other both under normal and disease conditions. For example, decrease in ATP generation due to oxidative stress causes defective Ca^2+^ pump function on plasma membrane leading to Ca^2+^ overload. High Ca^2+^ levels may cause cell death by forming permeability transition pore and collapse of transmembrane potential, which releases cytochrome c [54]. Excessive Ca^2+^ can lead to degradation of structural and enzymatic proteins by activating Ca^2+^ binding calpain proteases [55]. Apart from rapid cell death, subtle changes in Ca^2+^ homeostasis coupled with aging can lead to significant consequences such as cognitive decline [56]. Although some of this ATP is provided by glycolysis, the majority is produced in mitochondria [57]. Every part of the neuron requires ATP, and mitochondria, being the major source of ATP, are abundant in neurons. Some mitochondria are retained in the soma, whereas others are trafficked along axons and dendrites to supply energy-hungry sites, including the presynaptic terminal and near the nodes of ranvier [58].

Mitochondria are highly dynamic organelles and undergo both transient and rapid morphological adaptations that are crucial for many cellular processes including mitochondrial functions as well as quality control. The classical definition of mitochondrial dynamics refers to its coordinated cycles of fission and fusion [59]. These processes not only maintain mitochondrial shape, distribution, and size within cells, but also involve mitochondrial transport, fission, fusion, and selective degradation. Mitochondrial dynamics play a crucial role in neuronal development and function [59,60]. Emerging studies suggest multifunctionality of proteins linked to mitochondrial dynamics and cross-talk between mitochondrial dynamics, biogenesis, quality control and trafficking pathways [61]. Mitochondrial fission is important for homeostasis, fusion events enable mitochondria to complement proteins, repair DNA, and for equal distribution of metabolites [48,62]. In neurons, defective mitochondrial fusion leads to swelling of these organelles and prevents them from entering into distal, smaller branches of neurons, causing their degeneration in axons and dendrites [63]. In the case of defective fission, it leads to failure in isolating damaged parts of mitochondria leading to their autophagic degradation, promoting potential neuronal apoptosis [64]. Ablating genes involved in mitochondrial dynamics leads to defects in brain development. For example, MFN2 (important for mitochondrial fusion) conditional knock out in cerebellum leads to smaller cerebella and motor defects [63]. Other studies reported importance of MFN2 as playing role in synapse formation in human induced pluripotent stem cells (iPSCs) [65]. Homozygous mutations in Opa1 (function in mitochondrial fusion) are associated with infantile-onset encephalopathy [66]. Association of DRP1 (role in mitochondrial fission) in neuronal development become apparent in the case of newborns with abnormalities in brain development and optic atrophy with a lethal dominant negative allele of DRP1 [67]. In developing brains, mitochondria assume an elongated morphology in neuronal stem cells but are fragmented in neuronal progenitor cells. In the adult hippocampus, mitochondria display a mixture of globular and tubular structures [68,69]. In developing neurons, mitochondria are concentrated at the growth cones, where they are required to satisfy the high metabolic requirements [70]. In addition to ATP synthesis, mitochondria also play an important role in regulating intracellular Ca^2+^ dynamics, which in turn influence growth cone extension and collapse [71]. Interestingly, mitochondrial fission and fusion are also important for mitochondrial genome stability, via their role in maintaining mitochondrial DNA copy number, distribution, and integrity. Furthermore, mitochondrial dynamics mitigate the deleterious effects of mutations in mitochondrial DNA by complementation with intact DNA from healthy mitochondria via fusion [72,73,74,75].

Neurons are highly polarized cells with a cell body, numerous short and thick dendrites, and a long thin axon. Distinct structural and functional domains of neurons have different metabolic demands and display very non-uniform mitochondrial distribution. In comparison to any other region of the neuron, the synaptic terminal contains more mitochondria, where they are required for powering neurotransmission through the production of ATP, and for maintaining Ca^2+^ buffering. Neuronal mitochondria are dynamic, undergoing bidirectional movement in sync with neuronal processes; about one-third of mature mitochondria in axons are motile. Long-distance axonal transport is supported by motor proteins and microtubules. Both anterograde and retrograde transportation of mitochondria occur more often during development and become less frequent with age [76].

For proper synaptic function, stationary mitochondrial presence is required at regions with high energy requirements. Mitochondrial distribution and transport are highly correlated with local energy changes and metabolic demands, which is regulated by synaptic activation [77]. Sustained synaptic activity causes elevation of intracellular Ca^2+^ levels, and increased Ca^2+^ levels further inhibit mitochondrial movement either by activating voltage dependent Ca^2+^ channels or N-methyl-D-aspartate (NMDA) receptors. Docked mitochondria serve as stationary power plants, and for sequestering intracellular Ca^2+^ to sustain Ca^2+^ homeostasis [78,79].

Human brain circuitry is comprised of trillions of neurons and quadrillion synapses, and this connectivity underlies all human perception, emotion, thought and behavior. Neural circuits in response to a variety of stimuli undergo extensive sculpting and rewiring; this process of changes in synaptic connections in response to experience is called synaptic plasticity [80]. During synaptic activation and long-term potentiation, many studies reported changes in mitochondria, including increased Ca^2+^ pump activity, altered mitochondrial energy production, and increased mitochondrial gene expression [81,82,83,84]. Pharmacological studies revealed that inhibition of mitochondrial activity results in impairment of synaptic potentiation and neurotransmission [85,86].

An early and essential step for the formation of neuronal circuits is the establishment of neuronal polarity. Depletion of mitochondria from cells prevents axon formation, and even maintaining cellular ATP levels does not rescue this effect, suggesting a mechanism other than the ATP-generating function of mitochondria [87].

Maintenance of an appropriate pool of healthy mitochondria throughout the life of a neuron is extremely important. The challenge for neurons is the maintenance of a large number of neuronal mitochondria that require constant rejuvenation, even though their residence is far removed from the soma. One way neurons overcome this is by axonal protein synthesis mediated by soma-to-axon delivery of mRNAs. Intra axonal translation is necessary for regulation of growth cones, persynaptic plasticity, and injury response [88]. The majority of proteins generated by axonal translation including CoxIV and Atp5g1 are normally targeted to mitochondria [89,90]. In the human substantia nigra, a single neuron has been estimated to generate 1 to 2 million striatal synapses; this indicates that each cell has ~2 million mitochondria, throughout the several meters of axons [91,92,93]. The functional properties and behavior of mitochondria vary in axons and dendrites. In cultured hippocampal neurons, axons have twice as many motile mitochondria in comparison to dendrites, whereas dendrites have a higher proportion of highly charged mitochondria that are metabolically more active [94,95].

## 3. Mitochondrial Dysfunction in Common Neurodegenerative Diseases

As a dynamic organelle, the mitochondrion constantly undergoes fusion, fission, and transport in a regulated fashion. Increasing evidence of alteration in trafficking and fusion–fission dynamics in AD, Parkinson’s disease (PD), Huntington’s disease (HD), and ALS have been shown. In AD brains and peripheral cells derived from patients, energy deficiency is the fundamental characteristic feature. A reduction in the activity of OXPHOS complexes I, III, and IV are reported in the platelets and lymphocytes of AD patients and postmortem brain samples [96,97]. DRP1, a protein involved in mitochondrial fission, is reported to be abnormally expressed in postmortem brain samples of AD patients [98]. In PD patients, a mild deficiency in the mitochondrial electron transport chain (Complex I) is reported in the substantia nigra, and the inhibition of complex 1 in a mouse model causes similar behavioral and neuropathological symptoms as PD [99]. Many PD-linked proteins including Parkin, α-synuclein, PINK1, DJ-1, and leucine-rich repeat kinase 2, are associated with either mitochondria or mitochondrial proteins [100]. HD is caused by an abnormal expansion of CAG repeats in the *HTT* gene. There is extensive evidence for defects in bioenergetics and mitochondrial dysfunction in HD; for example, weight loss despite required caloric intake, increased lactate in the cerebral cortex and basal ganglia when observed using NMR spectroscopy, and reduced activity of the OXPHOS complexes II and III observed in cortical biopsies obtained from patients with both adult and juvenile onset HD. Besides, the number of mitochondria in HD postmortem brain tissue is reduced in mutant *Htt* knock-in mice [101].

## 4. Impaired Mitochondrial Dynamics and Plasticity in MND Pathogenesis

Many proteins linked to both familial and sporadic ALS and FTD are shown to be associated with mitochondria, including FUS, TDP-43, SOD1, and C9ORF72. Furthermore, the interactions of these proteins with mitochondria appear to be a key step in mitochondrial damage associated with ALS and FTD.

**Figure 1:**
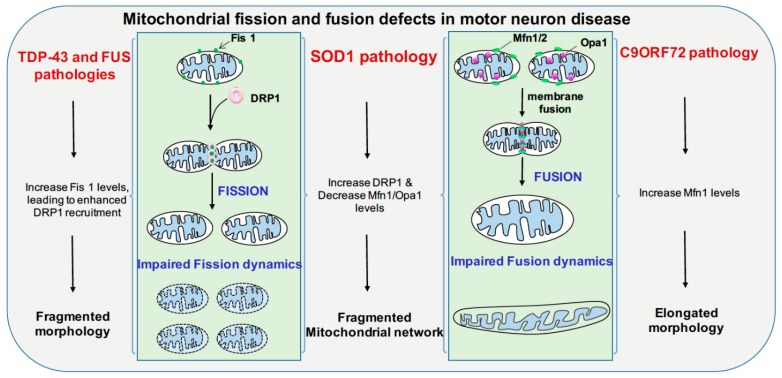
Mitochondrial fission and fusion defects in motor neuron disease and plausible mechanisms. Mitochondrial fusion is maintained at outer mitochondrial membrane by Mfn1/2 and Opa1 mediates inner mitochondrial membrane fusion. Mutated C9ORF72 affects mitochondrial fusion by increasing Mfn1 levels leading to elongated mitochondria, mutated SOD1 affects both fusion and fission by causing increase in DRP1 levels and decreasing Mfn1 and Opa1 levels, contributing to fragmented mitochondrial morphology. TDP-43 and FUS pathology cause increase in Fis1 levels leading to fragmented mitochondrial morphology. Fis1: Mitochondrial fission 1 protein, Mfn1/2: Mitofusin1 and 2, Opa1: Opa1 Mitochondrial dynamin-like GTPase, DRP1: Dynamin-1-lrelated protein, TDP-43: TAR DNA-binding protein 43, FUS: Fused in sarcoma, SOD1: Superoxide dismutase 1.

The pathology of TDP-43 is comprised of ubiquitination, hyperphosphorylation, and proteolytic cleavage of TDP-43, which leads to 35-kDa and 25-kDa insoluble aggregates, which are major components of inclusion bodies [102]. In cultured neural stem cells (NSC34 line), overexpression of full-length TDP-43 or its C-terminal fragment (25 kDa) induces mitophagy. In some mouse models of TDP-43, distinct changes in mitochondrial dynamics and aggregation are reported [103,104]. Mitochondrial dynamics are coordinated by regulators of fusion, which include mitofusin 1 and 2 (MFN1 and MFN2), along with dynamin-related protein 1 (DRP1), which is a regulator of fission [105]. Overexpression of either wild-type TDP-43 or the mutant form causes impairment of mitochondrial morphology and movement; overexpression of MFN2, which forms a complex with TDP-43, is shown to rescue TDP-43-induced mitochondrial dysfunction [106]. Selective expression of TDP-43 in the cortex and hippocampus of 4-month-old mice resulted in an increase in phosphorylation on the serine at position 637 of the DRP1 protein. This causes a decrease in fission and underscores a role of TDP-43 in fission dynamics (Figure 1) [107].

**Figure 2:**
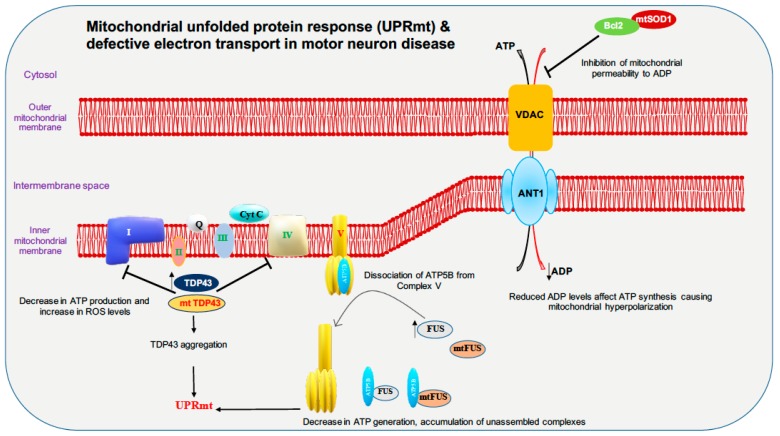
Motor neuron disease factors are linked to mitochondrial unfolded protein response (UPRmt) and defective electron transport chain. For ATP generation, proper functioning of electron transport chain is important. Mutated SOD1 (mtSOD1) was shown to cause inhibition of VDAC channel leading to polarity defects. FUS and TDP-43 pathology were reported to inhibit electron transport chain leading to decrease in ATP production and UPRmt. Accumulation of both wild-type FUS and the amyotrophic lateral sclerosis (ALS)-associated P525L mutant disrupts the formation of ATP synthase complex and suppresses the activity of ATP synthase by interacting with ATP5B, leading to loss of mitochondrial cristae, and thereby causing mitochondrial fragmentation. ATP5B: ATP synthase subunit beta, VDAC: Voltage-dependent anion-selective channel protein, ANT1: ADP/ATP translocase 1.

**Figure 3:**
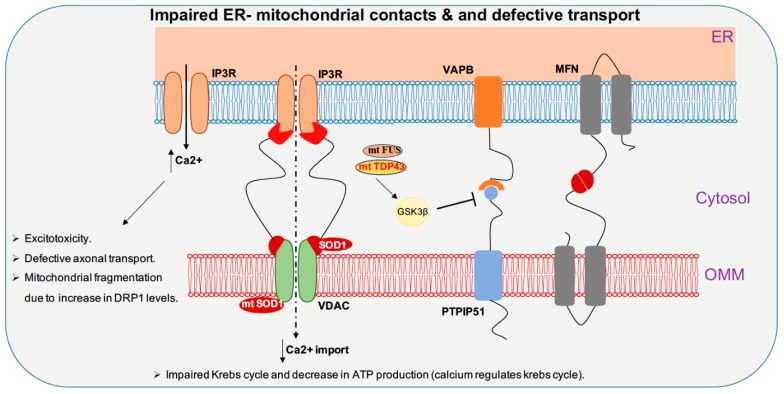
TDP-43, FUS, and SOD1 toxicities cause impaired Endoplasmic reticulum- mitochondrial contacts and defective mitochondrial transport. Exchange of Ca^2+^ between ER and mitochondria is important for maintaining Ca^2+^ homeostasis. Mutant FUS and TDP-43 pathology activate GSK3β, which inhibits VAPB-PTPIP51 interaction leading to decrease in contact between ER-mitochondria. SOD1 mutant inhibits VDAC channels and inhibit mitochondrial import of Ca^2+^. Contact disruption leads to increase in cytoplasmic Ca^2+^ levels which leads to disruption of various cellular pathways including mitochondrial transport. IP3R: Inositol triphosphate receptor, VAPB: Vesicle-associated membrane protein-associated protein B/C, MFN: Mitofusin, VDAC: Voltage-dependent anion channel, PTPIP51: Protein tyrosine phosphatase interacting protein 51, GSK3β: Glycogen Synthase Kinase 3 Beta, ER: Endoplasmic reticulum, OMM: Outer mitochondrial membrane.

Full-length TDP-43 is shown to be localized to the mitochondrial fraction upon stress in autosomal dominant ALS. In mitochondria, both wild-type and mutant TDP-43 bind preferentially to the mitocondrial-transcribed mRNAs ND3 and ND6, which encode subunits of complex I and inhibit oxidative phosphorylation (Figure 2). Surprisingly, suppression of TDP-43 localization to the mitochondria abolishes the toxicity of mutant and wild-type TDP-43 in mitochondria and neurons in ALS transgenic mice, and resulted in improved motor function [108]. In contrast, studies in TDP-43 A315T mutant mice and TDP-43 mutant fibroblasts revealed no impairment in mitochondrial bioenergetics [109]. Increased TDP-43 expression, due to either mutation or cellular stresses, causes the activation of the mitochondrial unfolded protein response (UPR^mt^) LonP1 which is a key mitochondrial protease involved in UPR^mt^ and plays an important role in the degradation of mitochondrial TDP-43. Downregulation of LonP1 expression in TDP-43 expressing flies results in more severe mitochondrial damage and advances disease onset [110]. Overexpression of wild-type and mutant TDP-43 affects endoplasmic reticulum (ER)-mitochondrial associations. TDP-43 activates glycogen synthase kinase 3β (GSK-3β), a kinase implicated in both ALS and FTD. Modulation of GSK-3β activity affects the Vesicle-associated membrane protein-associated protein B/C-Protein tyrosine phosphatase interacting protein 51 (VAPB-PTPIP51) interaction, inhibition of GSK-3β causes an increase whereas overexpression of GSK-3β causes a decrease in the VAPB-PTPIP51 interaction. Ca^2+^ homeostasis is affected by TDP-43 inhibition of the VAPB-PTIP51 interaction, leading to a decrease in mitochondrial Ca^2+^, which is essential for many pathways ranging from ATP synthesis to mitochondrial biogenesis (Figure 3) [111,112,113]. Ca^2+^ dysregulation caused by miscommunication between the ER and mitochondria may be considered to be a primary cause of motor neuron death in ALS [114].

FUS is reported to alter mitochondrial dynamics in various studies; an electron microscope study of two spinal cord samples of ALS-FUS reported that the disorganization of mitochondria and the ER in one sample corresponded to the P525L mutation [115]. Expression of ALS-associated FUS mutants R521G or R521H in the cytoplasm are associated with shortened mitochondria in cultured motor neurons [116]. Motor neurons derived from iPSCs of ALS patients harboring FUS R521H and P525L exhibited axonal transport defects, progressive ER-mitochondrial vesicle transport defects, and reduction in mitochondria-associated membranes. These defective phenotypes are shown to be rescued by the use of HDAC6 inhibitors or by genetically knocking down HDAC6 [117]. Although it was shown previously that the TDP-43-FUS complex regulates the mRNA expression of HDAC6, no difference in HDAC6 expression is reported in motor neurons derived from FUS mutant iPSC line. Both wild-type and mutant (P525L) FUS interact with HSP60. Elevated HSP60 expression was seen in brain tissue specimens from two out of three FTD-FUS patients. Knocking down HSP60 led to a reduction in mitochondrial FUS levels in cultured cells. Increased localization of FUS to mitochondria, due to either cellular stresses or pathogenic FUS mutations, leads to moderate increase in Fis1 levels leading to mitochondrial fragmentation, loss of membrane potential, increase in reactive oxygen species (ROS) production and defective mitochondrial axonal transport [118]. FUS, when accumulated inside mitochondria, interacts with mitochondrial ATP synthase catalytic subunit ATP5B and reduces mitochondrial ATP synthesis. Accumulation of both wild-type FUS and the ALS-associated P525L mutant disrupts the formation of ATP synthase complex and suppresses the activity of ATP synthase, leading to loss of mitochondrial cristae, and thereby causing mitochondrial fragmentation (Figure 2) [119]. Expression of both wild-type and ALS-associated mutant FUS disrupts ER-mitochondrial associations. FUS activates GSK-3β which is a known regulator of the ER-mitochondria association, which causes reduction of the interaction between VAPB and PTPIP51 (Figure 3) [120].

In primary cortical and motor neuron cultures, the ALS SOD1 G93A mutation selectively reduces mitochondrial anterograde transport [121]. Transgenic mice and rats with the SOD1 G93A mutation display defective anterograde and retrograde axonal transport of mitochondria, which is correlated with reduced levels of the outer mitochondrial membrane protein Miro1, a master regulator of mitochondrial axonal transport in response to cytosolic Ca^2+^ levels. The ALS SOD1 mutant inhibits axonal transport of mitochondria by Miro1 degradation, by inducing the Parkin/PINK1 dependent pathway [122]. SOD1 G93A forms high molecular weight aggregates and causes elevated oxidative lipid and protein damage in mitochondria; impairment of Ca^2+^ handling is also a common feature. These types of mitochondrial damage activate the PINK1/Parkin pathway, which halts mitochondrial transport [123]. The spinal cords of SOD1 G93A transgenic mice show progressively low levels of Mfn1 and Opa1, which are important for fusion; however, the levels of activated Drp1 and Fis1, which are important for fission, remain stable, thus disrupting the balance between fission and fusion (Figure 1) [123]. Mutant SOD1 forms a complex with BCL-2, binds to the cytoplasmic-facing domain of VDAC1, a mitochondrial porin located on the outer membrane of mitochondria that is important for metabolic crosstalk between mitochondria and rest of the cell. Binding of misfolded SOD1 to VDAC1 leads to inhibition of conductance (Figure 3) [124]. Reduced activity of respiratory chain complexes II and IV are observed in NSC34 cells expressing the SOD1 mutants G93A and G37R [125]. In G93A spinal cord mitochondria, impaired Ca^2+^ handling occurs very early on in the course of disease, and precedes respiratory chain defects, whereas in G85R hSOD1 expressing transgenic mice, Ca^2+^ capacity impairment occurs in the absence of oxidative phosphorylation dysfunction (Figure 2). This suggests that impairment of Ca^2+^ capacity may occur upstream of the cascade of events that leads to mitochondrial dysfunction in familial ALS [126].

Poly (GR), which results from repeat expansion in C9ORF72, favorably binds to mitochondrial ribosomal protein and compromises mitochondria function, which further leads to increased oxidative stress in neurons [127]. An altered balance between fission and fusion processes is evident in ALS and FTD patient fibroblasts carrying C9ORF72 mutation (mtC9ORF72) showed increased MFN1 levels and alterations in mitochondrial morphology (Figure 1) [128]. In mtC9ORF72 fibroblasts, the median mitochondrial membrane potential value increased significantly, suggesting hyperpolarized mitochondria. An increase in mitochondrial DNA content and mass is seen in mtC9ORF72 fibroblasts. Fragmentation of the mitochondrial network, deformation, and loss of mitochondrial cristae is observed in fibroblasts of ALS patients with repeat expansions [129]. In motor neurons derived from C9ORF72-associated ALS patient iPSC lines, swollen mitochondria were reported. Altered mitochondrial morphology and structural abnormalities in cristae are seen in 88% of C9ORF72 neurons in comparison to 2% of healthy neurons [128,130].

## 5. Mitochondrial Genome Instability in MND: Potential Role of TDP-43, FUS, and C9ORF72

Mitochondrial biogenesis and function require coordinated action of both nuclear and mitochondrial genomes. Mammalian mitochondria DNA is a circular molecule with 16,569 base pairs and can represent approximately 1% of total DNA in some cells. Mitochondrial DNA replication is not limited to the S phase but can occur throughout the cell cycle. Similar to nuclear DNA, mitochondrial DNA is constantly exposed to both external and internal DNA damaging agents, but mitochondrial DNA is generally more susceptible to DNA damaging agents as it is not protected by chromatinization and also lack some DNA repair factors [131]. Mitochondrial DNA is modified by oxidation and alkylation ~10 fold more than nuclear DNA [132,133,134,135]. In addition, hydrolytic damage, adduct formation, mismatched bases, and formation of single- and double-stranded breaks are reported in mitochondria [131,136,137,138]. Various DNA repair pathways have been reported in mitochondria, including base excision repair (BER), translesion DNA synthesis (TLS), homologous recombination (HR), non-homologous end joining (NHEJ), microhomology-mediated end joining (MMEJ), and the novel mismatch repair (MMR) pathway distinctive from nuclear MMR [139,140,141,142,143,144,145,146], although the level of proficiency of each of these pathways is not known. The nucleotide excision repair (NER) and Fanconi anemia (FA) pathways are not reported in mitochondria, but evidence of mitochondrial localization of multiple proteins involved in these pathways has been reported (Table 1) [147,148].

The ALS proteins TDP-43, FUS, and C9ORF72 were recently shown to be important for maintaining nuclear genomic stability due to their involvement in DNA damage repair. For example, FUS is recruited to the DNA damage site in a PARP1-dependent manner, where it recruits XRCC1/LIG3 and is required for efficient function of LIG3 [31]. Our laboratory identified TDP-43 as a key component of NHEJ-mediated DNA double-strand break repair machinery, where it acts as a scaffold for the recruitment of XRCC4-LIG4 [149]. Furthermore, C9ORF72 iPSC differentiated motor neurons show increased oxidative stress and DNA damage in an age-dependent manner [129]. Although the roles of these proteins in mitochondrial dynamics and plasticity have been explored, their emerging DNA damage response role in the nuclear genome raises the question of whether these proteins are also important for the maintenance of genomic stability in mitochondria. It is important to note that mitochondria have a limited number of back up DNA repair pathways, unlike the nucleus. For example, LIG3 (mitochondrial isoform) is the only ligase in mitochondria that supports both its genome replication and repair. Furthermore, although XRCC1 acts as a scaffold for LIG3 in the nuclear genome, studies have reported that XRCC1 is not localized in mitochondria [150]. This raises the question of whether FUS replaces XRCC1 as a stabilizing scaffold for mitochondrial LIG3. Furthermore, PARP-1 also localizes to mitochondria, whereas the role of PARylation in mitochondrial genome repair is still emerging [121,122,123]. Interestingly, recent studies suggested that XRCC4 may localize to mitochondria, where it may collaborate with LIG3 for mitochondria DNA double-strand break repair [151]. In view of our recent discovery of TDP-43′s association with XRCC4, it is likely that TDP-43 function in mitochondrial DNA repair by recruiting the XRCC4-LIG3 complex [149,151]. The potential role of TDP-43 and FUS in maintaining mitochondrial genomic stability requires further investigation.

## 6. Effect of Mitochondrial Dysfunction beyond Mitochondria in the CNS

Although mitochondria act as major source of energy, other functions performed by these organelles are equally important for the cell. In the CNS, mitostasis assumes greater importance, because the brain consumes a significant amount of oxygen, and thus generates higher levels of ROS, which are reported in many neurological diseases including ALS, AD, PD, and dementia [185,186]. Ca^2+^ homeostasis is also important for various CNS functions including neurotransmission, along with ER-mitochondrial function, in regulating Ca^2+^ homeostasis.

Mitochondrial oxidative phosphorylation is believed to be a major source of cellular ROS in most cell types [187]. Particularly in neurons, mitochondria act as a primary site of ROS generation. Outside mitochondria there are other enzymes, which are associated with ROS generation including NADPH oxidase, neural nitric oxide synthase and monoamine oxidase [188,189,190,191,192]. In moderate or low levels, ROS are important for neuronal function and development, but excessive levels are toxic [193,194]. Oxidative stress plays an important role in the development of various diseases ranging from cancer to autoimmune disorders. With increasing age, the redox system becomes imbalanced, leading to elevated levels of reactive oxygen and nitrogen species. It has been shown that different regions of the brain have different levels of vulnerability for oxidative stress. For example the hippocampus, cerebellar granule cells, and amygdala have been shown to be most susceptible to oxidative stress, and are consequently the first to undergo functional decline [195]. Neurons, which carry out dopamine metabolism, are more susceptible to ROS-induced damage; this is also the case for neurons with high metal content [196]. The precise chain of events occurring within the CNS that leads to oxidative stress-induced cognitive or behavioral decline needs to be understood at multiple levels. Apart from affecting several essential mitochondrial functions, including various metabolic functions such as fatty acid oxidation, heme synthesis, amino acid metabolism, and the urea cycle, oxidative damage increases the mitochondrial tendency to release cytochrome c, leading to activation of apoptosis [197].

Overexcitation of NMDA receptors by glutamate is thought to be a key driver of neuronal damage after stroke or neurodegenerative diseases. NMDA receptor activates neurotoxicity by activating nitric oxide synthase which results in nitric oxide (NO) production. Furthermore, peroxynitrites are formed in mitochondria due to presence of superoxides; these lead to DNA damage and PARP-1 activation, which plays a key role in NMDA excitotoxicity [198]. Apoptosis-inducing factor (AIF) is normally localized in mitochondria; following PARP-1 activation, AIF translocates to the nucleus, triggering nuclear shrinkage, chromatin condensation, and DNA fragmentation [199].

Physical and functional coupling between the ER and mitochondria plays an important role in a variety of cellular pathways, and alterations in this coupling are associated with several diseases, including ALS, AD, and PD [200,201]. The ER-mitochondria contact is important in multiple pathways including cell proliferation, death, autophagy, lipid metabolism, Ca^2+^ signaling, the unfolded protein response, inflammation, and bioenergetics. Motor neurons have multiple properties that make them more vulnerable to Ca^2+^ dysregulation when compared to other neuronal populations. They have a high number of AMPA receptors which are permeable to Ca^2+^ at the postsynaptic terminal, resulting in greater vulnerability to excitotoxicity during excitatory neurotransmission events [202]. Motor neurons have decreased cytosolic buffering capacity because they express low levels of Ca^2+^ buffering proteins such as calbindin and parvalbumin, which render them more dependent on mitochondrial Ca^2+^ buffering [203,204]. The Ca^2+^ level in mitochondria regulates ATP production by activating rate limiting enzymes of the Krebs cycle. Decreased ATP levels directly impact the axonal transport of mitochondria and other vesicles important for neuronal function. Elevated cytosolic Ca^2+^ levels due to defective ER-mitochondria function leads to disruption of mitochondrial axonal transport. Excessive Ca^2+^ interacts with Miro 1 and prevents its association with mitochondrial KIF5, leading to defects in axonal transport and reduced local ATP levels [79,205].

In ALS, over expression of both wild-type and mutated TDP-43 leads to a decrease in ER-mitochondria physical and functional coupling. TDP-43 perturbs the interaction between the outer mitochondrial membrane protein, protein tyrosine phosphatase-interacting protein-51 (PTPIP51), and resident ER protein vesicle-associated membrane protein associated protein-B (VAPB) by activating GSK-3β (Figure 3) [111]. FUS overexpression is associated with impairment of ER-mitochondria juxtaposition, Ca^2+^ transfer, and mitochondrial ATP production [120]. Over expression of the ALS-associated SOD1 G93A mutation leads to altered Ca^2+^ homeostasis. Taken together, these studies indicate that alteration of ER-mitochondrial functional coupling could be an early event in ALS and FTD [126]. Apart from neuronal diseases, alterations in ER-mitochondrial interactions have been shown to be associated with metabolic defects such as obesity and insulin resistance. Ca^2+^ acts as a mechanistic link between metabolic impairment and ER-mitochondrial interaction. Increasing evidence has been reported on the ability of cancer cells to remodel their intracellular Ca^2+^ signaling, as it favors their survival and proliferation. Modulation of ER-mitochondrial Ca^2+^ crosstalk enhances resistance to apoptosis [206].

## 7. Conclusions and Perspectives

Although MNDs were first reported more than a century ago, there is yet no effective treatment. Two drugs, namely Riluzole and Edaravone, are currently approved by FDA for use in ALS. The mechanism of action of Riluzole is not very clear, but some studies point to its antioxidant activity by induction of glutathione synthesis, which can only extend survival by 3 months. Edaravone acts as a free radical scavenger [207]. Overwhelming evidence suggests that mitochondrial dysfunction plays a significant role in proteinopathies of neurodegenerative diseases. In ALS, TDP-43 and FUS are reported to be recruited in mitochondria; toxic or mutant forms of these proteins lead to defects in mitochondrial function and mitostasis. Although recent studies provide some insights into how these proteins may affect mitochondrial function, a comprehensive understanding of how these proteins are recruited to mitochondria, their differential regulation in normal versus affected neurons, and the physiological as well as pathological importance of these proteins in mitochondria are required to develop effective mechanism-based therapeutics. Many of the strategies used to develop therapeutic interventions against imbalances in mitochondria focus on antioxidant therapy. Metabolic intermediates such as creatine and Coenzyme Q10, although promising in animal studies, were not effective in clinical trials [208,209,210,211]. Many drugs and gene therapy-based approaches, targeted to improve mitochondrial function, have also not shown promise in trials. Free radical scavengers such as Ederavone only had a marginal effect on disease progression. The inconsistency between mouse models and clinical trials raises an essential need to generate clinically relevant disease models [212,213]. Furthermore, there are variations in the type of mitochondrial dysfunction caused by each protein, suggesting that a single form of therapy will not effectively attenuate all these different aspects. Thus, it is important to identify critical pathways and mechanisms of mitochondrial dysfunction during disease initiation and progression. Future studies should also focus on common mechanisms among various neurodegenerative conditions, which will enhance our understanding of essential requirements for neuronal survival.

## Figures and Tables

**Table 1:** DNA repair proteins and their associated pathways reported to be localized in mitochondria.

DNA Repair Pathway	Target Lesions	Repair Protein(s) Localized in Mitochondria
Base excision repair (BER)	Base modification	APE1 [152], APE2 [153], APTX [154], DNA2 [155], FEN1 [156], LIG3 [157], MPG [157], MUTYH [158], NEIL1 [159], NEIL2 [160], NTHL1 [161], NUDT1 [162], OGG1 [163], PARP1 [164], PNKP [160], POLB [165], POLG [166], TDP1 [167], UNG [168].
Translesion synthesis (TLS)	Lesion causing replication fork stalling	POLZ [169], POLQ [151].
Homologous recombination (HR)	Double-strand breaks containing strong sequence homology	RAD50 [170], RAD51 [171], MRE11 [172], NIBRIN [170], LIG3 [157].
Non-homologous end joining (NHEJ)	Double-strand breaks with little or no sequence homology/blunt ends	ATM [173], ATR [174], KU80 [175], XRCC4 [151].
Microhomology-mediated end joining (MMEJ)	Double-strand breaks with micro homology 3–25 bp in length	LIG3 [150], PARP1 [164], CtIP [145], MRE11 [172], RAD50 [170].
Mismatch repair (MMR)	Sequence mismatches	HMBG1 [176], MLH1 [177], YB-1 [178].
Nucleotide excision repair	Helix distorting and bulky lesions	CSA [147], CSB [147], LIG3 [157], ERCC2 [179].
Fanconi anemia pathway (FA)	DNA interstrand cross links	FANCC [148], FANCD2 [180], FANCG [181], FANCM [182], FANCO [183], FANCS [184].
